# Abundant dynamics of group velocity locked vector solitons from Er-doped fiber laser based on GO/PVA film

**DOI:** 10.1515/nanoph-2022-0544

**Published:** 2022-10-28

**Authors:** Ja-Hon Lin, Zhan-Yao Zhang, Zhen-Ying Li, Peng-Chun Peng, Yu-Feng Song, Han Zhang

**Affiliations:** Advanced nanophotonics technology laboratory, Department of Electro-Optical Engineering, National Taipei University of Technology, Taipei 10608, Taiwan; College of Electronics and Information Engineering, Shenzhen University, Shenzhen 518060, P. R. China; International Collaborative Laboratory of 2D Materials for Optoelectronics Science and Technology of Ministry of Education, Institute of Microscale Optoelectronics, Shenzhen University, Shenzhen 518060, P. R. China

**Keywords:** dual wavelength, graphene oxide, group velocity locked vector solitons, Lyot filter, multiple soliton

## Abstract

With the insertion a segment of polarization-maintaining fiber (PMF) inside the cavity, abundant dynamics of group velocity locked vector solitons (GVLVSs) in Er-doped fiber laser have been investigated by using graphene oxide/polyvinyl alcohol (GO/PVA) film as a saturable absorber (SA). The generated Kelly sidebands in emission spectra reveal peak-valley or valley-peak alternation and slightly shift in two orthogonal components, which are the characteristics of GVLVSs. Through proper adjustment of polarization controllers (PCs) inside the EDFLs cavity, versatile vector soliton dynamics such as polarization locked GVLVSs (PL-GVLVSs), polarization rotation GVLVSs (PR-GVLVSs), dual wavelength GVLVSs, bound state GVLVSs, bunch GVLVSs and harmonic mode-locking GVLVSs (HML-GVLVSs) have been observed. The separation between two emission peaks from the dual wavelength GVLVSs was controlled by the Lyot filter and related to the insertion length of PMF inside the cavity. Unlike PL-GVLVSs, the period-doubling phenomenon has been found in two orthogonal components of the PR-GVLVSs. Besides, the bound state GVLVSs were generated showing strongly modulated interference fringes in emission spectrum. For the bunch and HML GVLVSs, the number of solitons inside the cavity increased with the pump power, and it showed the quintuple solitons and the 7th HML-GVLVSs at the highest pump power.

## 1 Introduction

Owing to their superior characteristics such as ultrashort pulsewidth and relatively high peak intensity, passively mode-locked fiber lasers (PML-FLs) have been widely used in various fields for specific applications such as micromachining, nonlinear optical measurement, biomedical imaging, light detection, ranging, and optical communications [1–4]. In order to produce mode-locked pulses, the artificial saturable absorbers (SAs), such as nonlinear polarization rotation (NPR) [5, 6] and nonlinear amplifier loop mirror (NALM) [7], have been adopted as an ultrafast optical switching inside the cavity. Besides, many researchers have tried to use the semiconductor saturable absorber mirror (SESAM) [8] and cost-effective two-dimensional (2D) nanomaterials, like graphene [9, 10], carbon nanotubes (CNTs) [11], topological insulators [12], and black phosphorous [13, 14] as material-based SAs to fabricate robust ultrashort pulsed lasers. Generally, the produced 2D nanosheets were mixed with polymers, like polyvinyl alcohol (PVA) and polymethyl methacrylate (PMMA), to form thin film SAs and were then sandwitched between two fiber ferrules. In addition, novel material like hydrazone organics [15], PbTe [16], and metal organic frameworks (MOFs) material like porous nickel oxide [17], porous dodecahedron rGO-Co_3_O_4_ [18], and CuO octahedra [19] have been integrated with microfiber or tapered fiber to produce SAs for the stabilized mode-locked pulse generation in Er-doped fiber lasers (EDFLs).

Based on the relatively complicated interaction between gain, loss, nonlinearity, and dispersion inside the cavity, PML-FLs have been recognized as an appropriate platform for the investigation versatile soliton dynamics [20], such as noise-like pulse [21], dark soliton [22], bound soliton [23], multiple soliton [24], harmonic mode-locking [25], and soliton rain [26]. Besides, vector solitons (VSs) have attracted great attention since the pioneer work by Menyuk [27, 28], who theoretically predicted the trapping of two orthogonal polarized solitons in a single mode fiber (SMF). In the following, polarization rotation VSs (PRVSs) [29, 30] and polarization-locked VSs (PLVSs) [31, 32] have also been theoretically proposed in weakly birefringent SMF. In the last decades, VSs have been experimentally investigated in PML-EDFLs based on the artificial SAs like the NALM in a figure eight configuration [7], in which the various vector nature of multi-soliton dynamics in combination with the PLVSs and the PRVSs was observed. Through the NPR, both vector and scalar solitons coexist within the laser cavity, depending on the local birefringence [33]. Besides, material-based SAs, such as niobium diselenide (NbSe_2_) and single-walled CNTs [11, 34], have been used to investigate PLVSs and PRVSs in PML-EDFL. On the other hand, graphene has also been inserted the inside cavity of EDFL to produce the bound state, multiple VSs, and harmonic mode-locking PRVSs [35–37].

Dual wavelength VSs are another fascinating phenomenon that has been reported in EDFL based on the little layer black phosphorus (BP) as SAs [38]. Song et al. [39] in 2020 reported dual wavelength VSs within net anomalous cavity dispersion through the dissipative mechanism induced by the effective gain bandwidth limitation. In 2008, Zhao et al. [40] experimentally observed and theoretically simulated the group velocity locked vector solitons (GVLVSs) in a weak birefringence PML-EDFL by using SESAM as an SA. For the compensation of group velocity mismatch on two orthogonal axes, the Kelly sidebands of GVLVSs reveal a relative shift in horizontal and vertical components by the self-phase modulation (SPM) and cross-phase modulation (XPM). After inserting a segment of polarization-maintaining fiber (PMF) inside the cavity to enhance birefringence, the compound of multiple soliton complexes, or GVLVSs molecules, has also been investigated [41].

Graphene oxide (GO) is an atomic layer of carbon bonded with oxygen functional groups, which can be easily dispersed and preserved in deionized (DI) water. The covalent oxygen functional groups in GO make it reveal remarkable hydrophilic property and provide noteworthy mechanical strength to offer superior flexibility and processability. Today, GO has been fabricated in various devices for certain applications including ultrafast photonics because of its ultrafast carrier relaxation and large optical nonlinearities. The GO-based SAs exhibit various advantages, including fast recovery time, easy of fabrication, and cost effectiveness that has been adopted to produce the near infrared (NIR) PML-FLs by using Yb-, Er-, and Tm-doped fiber as gain medium [42–44]. Compared to other SAs, the produced graphene oxide/polyvinyl alcohol (GO/PVA) film reveals a robust mechanical property and thermal stability that has been used to produce ultrashort pulsed lasers in EDFL with a wide wavelength tuning range and long-term stability [45]. However, the study of VSs based on the few layer GO is rare. In order to investigate the abundant dynamics of GVLVSs in PML-EDF, the cost effective GO/PVA film was adopted as SAs. In addition, a certain length of PMF has been adopted to produce the dual wavelength VSs in the C band. Through proper adjustment of the polarization controllers (PCs) inside the cavity, we investigated versatile dynamics including polarization locked, polarization rotation, bound state, bunch, and harmonic mode-locking GVLVSs.

## 2 Sample preparation and characterization of GO/PVA film

**Figure 1:**
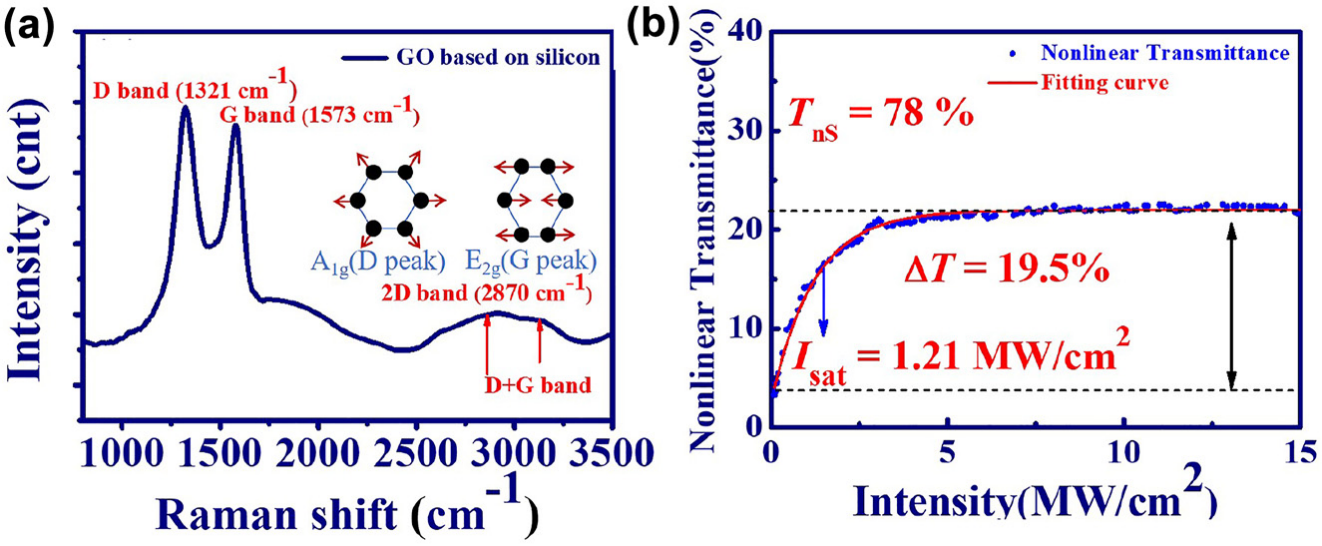
Characterization of GO/PVA film. (a) Raman spectrum of the GO platelet. (b) Nonlinear transmittance of the GO/PVA film (blue circle) and the theoretical fitting curve (red solid line) by the nonlinear transmittance.

The GO nanoplates were produced by the liquid phase exfoliation. First, GO powder and sodium dodecyl sulfate (SDS) were dissolved into deionized (DI) water and then ultra-sonification for about 2 h. After exfoliation, the GO suspension was centrifuged with a rotation speed of 16,000 rpm for 10 h. Here, the bottom layer of suspension is selected and then homogeneously mixed with the PVA solution by the magnetic stirrer for several hours. Finally, the GO/PVA dispersion was poured into a plastic mold and dehydrated in an oven for two days. In Figure 1(a), the Raman spectrum of the GO nanoplates [46] reveals two main peaks, i.e., the D and G bands. The D peak of GO around 1321 cm^−1^ resulted from a defect-induced breathing mode of sp^2^ rings. The G peak of GO around 1573 cm^−1^ is due to the first order scattering of the *E*_2g_ phonon of sp^2^ carbon atoms. In Figure 1(b), the nonlinear transmittance of the GO/PVA film was measured by the homemade PML-EDFL as a light source with the central wavelength at 1558 nm. Through the theoretical fitting of nonlinear transmittance (red curve) [47], the parameters of modulation depth (Δ*T* = 19.5%), nonsaturable transmission (*T*_*ns*_ = 78.0%) and saturation intensity(*I*_sat_ = 1.21 MW/cm^2^) have been obtained.

## 3 Experimental results and discussions

**Figure 2:**
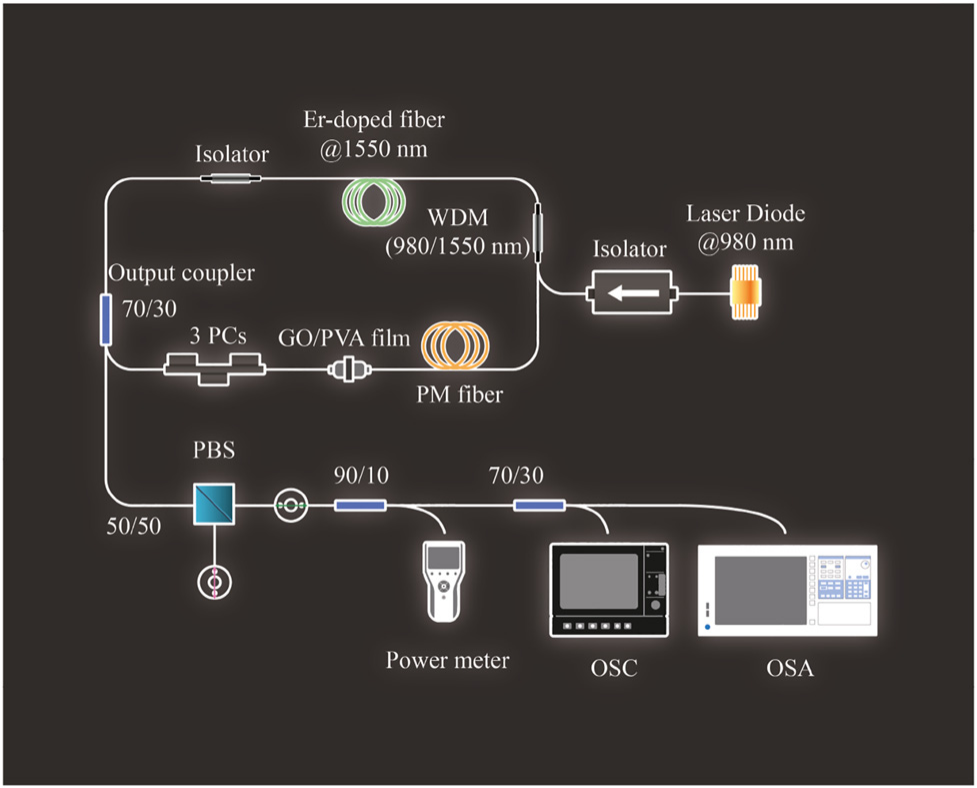
Experimental setup of ring configuration EDFL. (EDF: erbium doped fiber, WDM: wavelength-division multiplexing, ISO: isolator, PC: polarization controller, SA: saturable absorber (GO/PVA film), PMF: polarization maintaining fiber, PBS: polarization beam splitter).

The experimental setup of ring cavity configuration EDFL is schematically depicted in Figure 2. It comprises a 0.65 m long Erbium-doped fiber (EDF, 110 dB/m @1550 nm, *β* ^2^ ∼ 0.128 ps^2^/m) as a gain medium. A 980 nm laser diode was used as a pump source and coupled into the cavity by the 980/1550 wavelength division multiplexer (WDM). A polarization insensitive isolator (PI-ISO) was used to make sure there was unidirectional propagation of pulsed light inside the cavity. The PCs were used to control the polarization of pulsed light inside the cavity. The mode-locking of the EDFL was based on a GO/PVA film, which was sandwiched between two fiber connectors. The output coupler (OC) of the laser was a 30/70 fiber coupler, in which 30% intra-cavity light was outputted. A segment of PFM (beat length (*L*_*b*_) < 4 mm @1300 nm, PM-1300XP) with lengths of 35 cm or 40 cm was inserted inside the cavity to produce the vector solitons. In order to observe the vector characteristics, a fiber-based polarization beam splitter (PBS) was used outside the cavity to obtain the emission spectrum and time trace on two orthogonal axes. An optical spectrum analyzer (OSA, AQ 6370 Yokogawa Inc.) was adopted to monitor the optical spectrum of the output signal with a resolution of 0.05 nm. The time trace and pulsewidth of mode-locked pulse trains were recorded by a 2 GHz oscilloscope (OSC, 620Zi, LeCroy Inc.) and intensity autocorrelator (Femtochrome FR-103XL).

**Figure 3:**
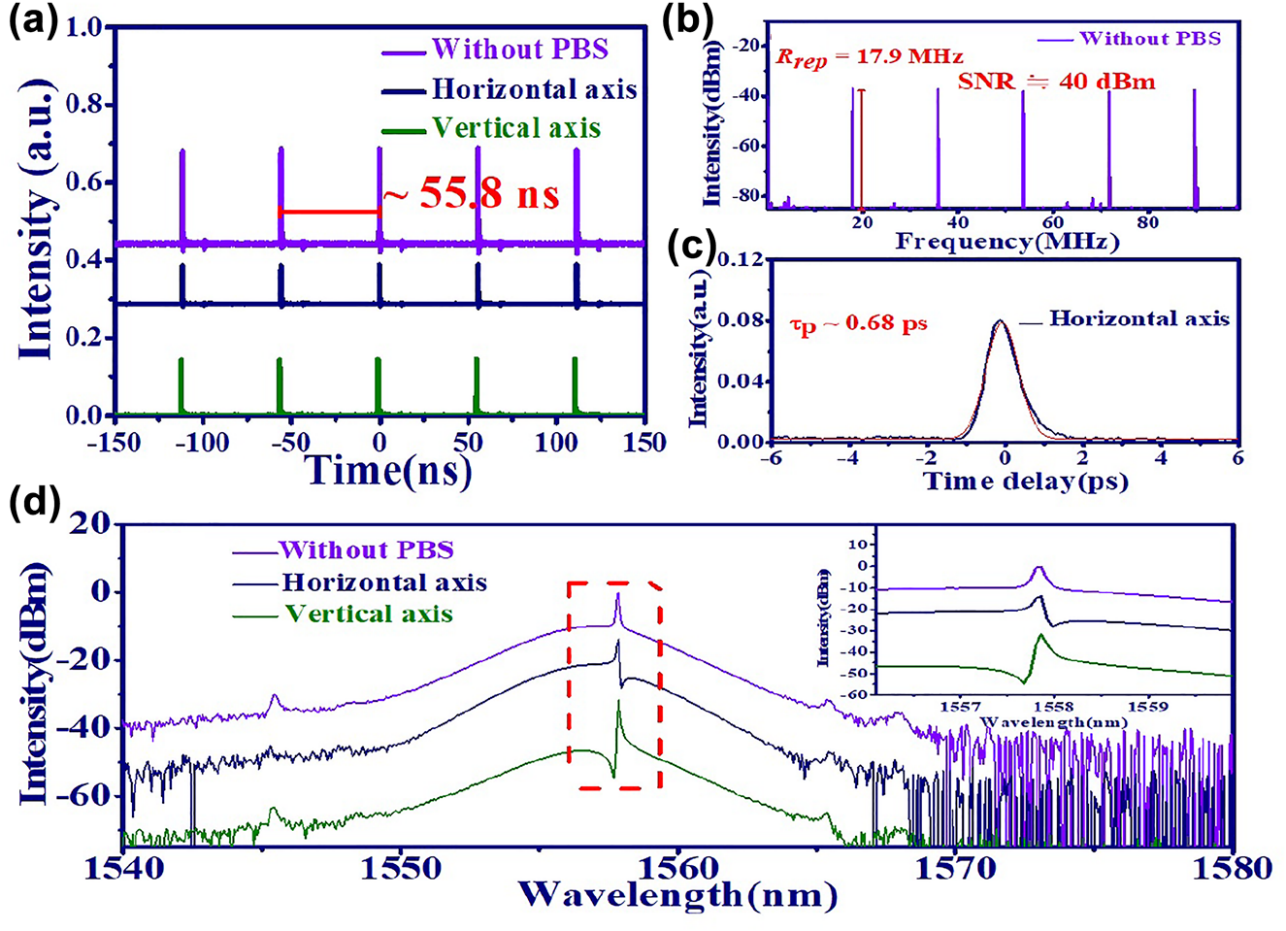
PL-GVLVS from EDFL based on GO/PVA film. (a) Time trace (without PBS: purple line, horizontal axis: navy line and vertical axis: green line), (b) RF spectrum without PBS, and (c) IAC trace on horizontal axis, and (d) optical spectrum and zoomed in spectrum of the Kelly sidebands (inset figure).

In previous reports, both PRVSs [29] and PLVSs [31] have been predicted in weakly birefringent SMF and experimentally observed in mode-locked fiber lasers [32]. The generation of PLVSs is attributed to the lock of the relative phase between two orthogonal components at ±*π*/2 by means of Kerr nonlinearity [32]. In Figure 3(a), the time trace of PLVSs indicates that the period of mode-locked pulses is around 55.8 ns, which is consistent with the cavity round trip time. In Figure 3(b), the radio-frequency (RF) spectrum of PML-EDFL without PBS (resolution of 30 KHz) shows that the corresponding repetition rate is around 17.9 MHz and the SNR is around 40 dBm. The pulse duration of soliton on the horizontal axis ∼680 fs is obtained by the interferometric autocorrelation (IAC) trace in Figure 3(c). In Figure 3(d), the corresponding optical spectrum illustrates that the Kelly sidebands of PLVSs reveal peak-valley and valley-peak variation in two orthogonal polarization components (inside the red dashed box in Figure 3(d)). Nevertheless, the Kelly sidebands of GVLVSs without PBS only show the peak in the optical spectrum. The zoomed in spectrum further shows that the peak wavelength of the Kelly sideband in two orthogonal components shifts slightly. It is recognized that the obvious spectrum difference in the two orthogonal polarized components is required to form these polarization locked GVLVSs [41].

**Figure 4:**
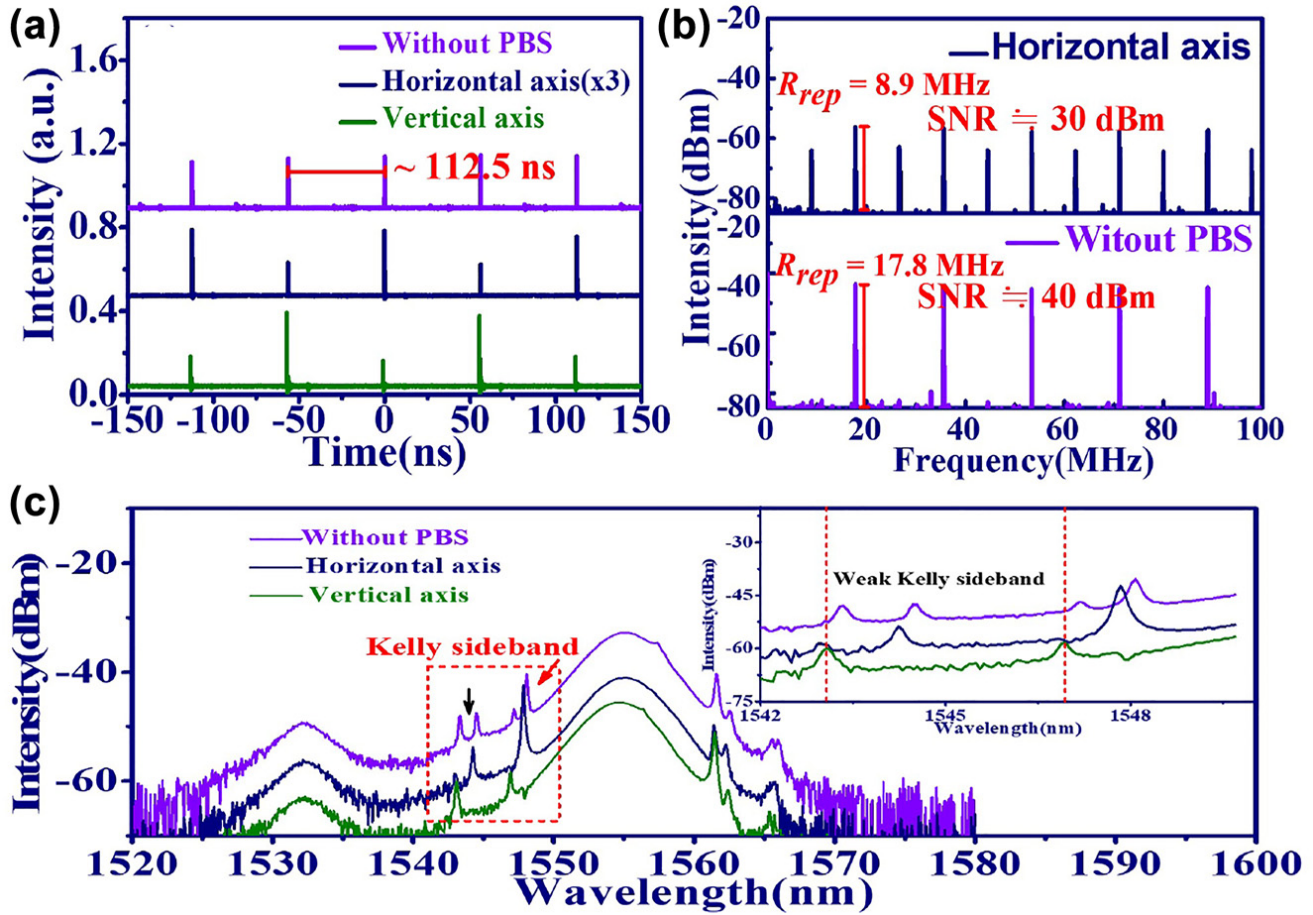
PR-GVLVSs from EDFL based on GO/PVA film. (a) Time trace, (b) RF spectrum, and (c) optical spectrum and zoomed in spectrum of Kelly sidebands (inset figure). (Without PBS: purple line, horizontal axis: navy line and vertical axis: green line).

In addition to the PLVSs, the PRVSs have also been observed in this work through the proper adjustment of the PCs as shown in Figure 4 (with insertion of a 31 cm long PMF). Similar to the previous report [48], the pulsed trains of PRVSs reveal period doubling in two orthogonal components (horizontal: navy line, or vertical: green line in Figure 4(a)) that can also be verified in their RF spectrum with the 8.9 MHz repetition rate at the top of Figure 4(b). However, the phenomenon of period doubling cannot be observed in the time trace of GVLVSs without PBS (purple line in Figure 4(a)) and shows the 17.8 MHz repetition rate in the corresponding RF spectrum in 4(b). Owing to the group velocity mismatch of VSs on the two orthogonal axes of the PMF, the shape and emission peaks from the two sets of Kelly sidebands inside the red dashed box varied slightly on the horizontal and vertical axes as shown Figure 4(c). The zoomed in spectrum in Figure 4(c) shows that one of two separated Kelly sidebands (without PBS) can only appear on either the horizontal or vertical axis.

**Figure 5:**
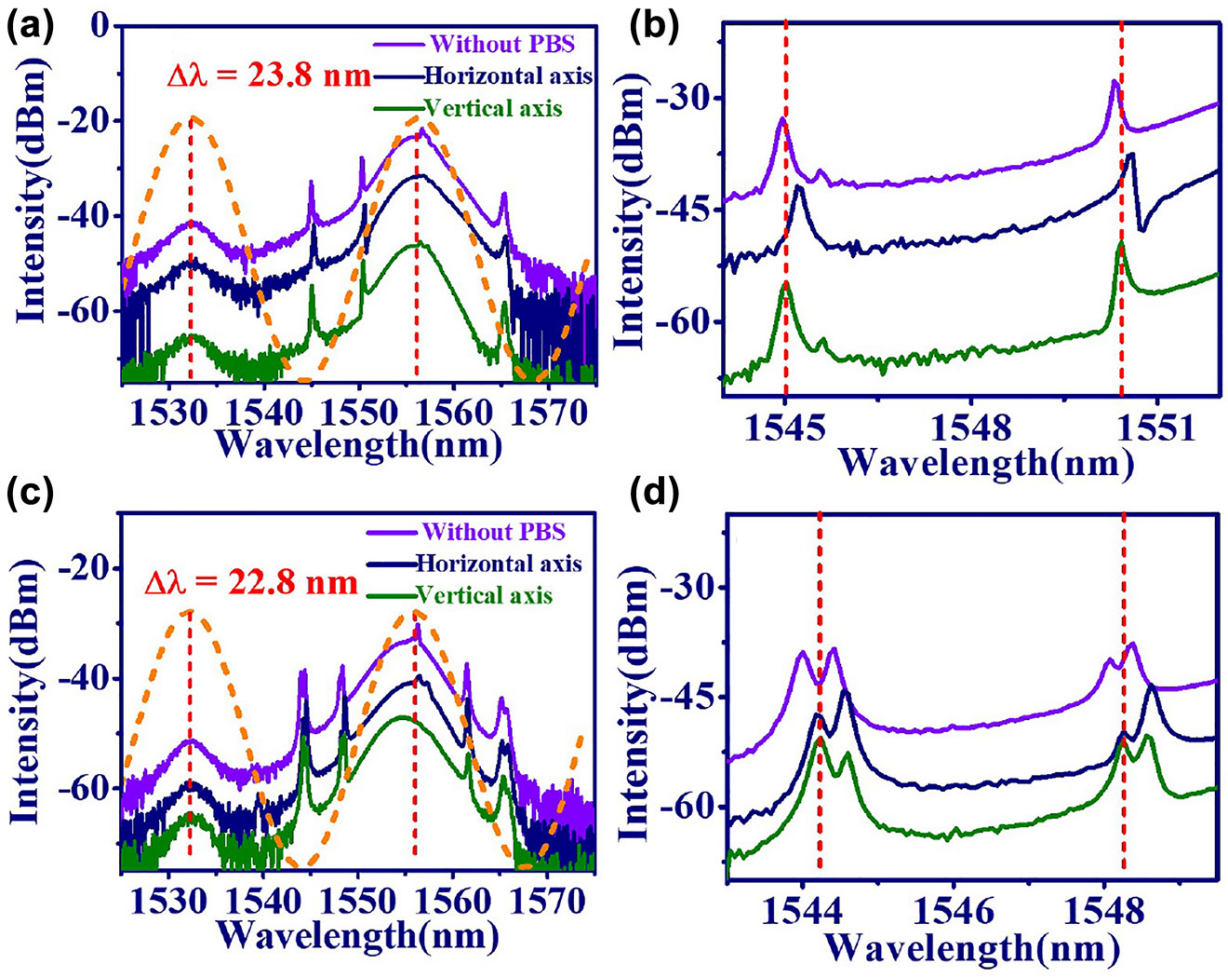
Dual wavelength GVLVSs from EDFL based on GO/PVA film. (a) Optical spectrum and (b) zoomed in optical spectrum of Kelly sidebands with 35 cm long PMF. (c) Optical spectrum and (d) zoomed in optical spectrum of Kelly sidebands with 40 cm long PMF (without PBS: purple line, horizontal axis: navy line, and vertical axis: green line). (Orange dashed line in (a) and (c) shows the estimated transmission spectrum of invisible filter by Eqs. (1) and (2)).

In this work, stable dual wavelength GVLVSs was produced in EDFL with insertion of a certain length of PMF inside the cavity, which plays the role of the Lyot filter [49, 50]. For the 35 cm long PMF inside the cavity, dual emission peaks around 1532.0 and 1555.8 nm were observed in the optical spectrum as shown in Figure 5(a). Here, the optical spectrum on the horizontal, vertical, and without PBS are plotted as navy, green, and purple lines, respectively. The zoomed in optical spectra of the Kelly sideband is shown in Figure 5(b). The emission peaks of the Kelly sideband on the horizontal axis reveal a slight red drift around 0.1–0.2 nm relative to the vertical axis for the compensation of the group velocity mismatch induced by the birefringence of PMF. As the length of PMF increases to 40 cm, the spacing (Δ*λ*) of the two emission peaks slightly shrinks to the 22.8 nm in Figure 5(c). The zoomed in optical spectrum in Figure 5(d) shows that extra sidebands emerge.

Theoretically, the separation of two transmission peaks (Δ*λ*) is controlled by the Lyot filter induced by the birefringence of fiber with the relation

(1) $$\Delta \lambda =\lambda_{0}^{2}/\left(BL\right),$$

where *λ*_0_ is the central wavelength, *L* is the length of PMF, and *B* = *λ*_0_/*L*_*b*_ is the birefringence. Considering Δ*λ* = 23.8 nm, the estimated beat length *L*_*b*_ of PMF is about 5.8 mm through Eq. (1). In addition to playing the role of ultrafast switching for the mode-locked pulse generation, the NPR can be regarded as the invisible filter [51], in which the transmission is estimated by

(2) $$\begin{array}{ll} {\left|T_{1}\right|}^{2} & ={cos}^{2}\theta_{1}{cos}^{2}\theta_{2}+{sin}^{2}\theta_{1}{sin}^{2}\theta_{2}\\ & \;+\frac{1}{2}sin2\theta_{1}sin2\theta_{2}cos\left(\Delta \phi_{L}+\Delta \phi_{NL}\right), \end{array}$$

where *θ*_1_ and *θ*_2_ are the azimuth angles of the polarizer and the analyzer relative to the fast axis of the fiber, and Δ*ϕ*_*L*_ and Δ*ϕ*_*NL*_ are the linear and nonlinear phase delays. Here, we only consider linear phase delays Δ*ϕ*_*L*_, which can be expressed as Δ*ϕ*_*L*_ = Δ*ϕ*_0_ + 2*π* (1 – Δ*λ*/*λ*_0_) *L*/*L*_*b*_, where Δ*ϕ*_0_ is the initial phase delay between the two orthogonal modes propagating in the fiber, *λ*_0_ is the central wavelength of the optical pulse, and Δ*λ* is the wavelength detuning against *λ*_0_. Considering the length of two PMFs with *L* = 35 and 40 cm, i.e., the ratio of *L*/*L*_*b*_ is 60 and 68, the estimated transmittance of invisible filter by Eq. (2) is shown by dashed line in Figure 5(a) and (c). Consistence with the experimental results, the interval of transmission peaks decreases as the value of *L*/*L*_*b*_ increases.

Multiple soliton operation is another well-known phenomenon that has been extensively studied in PML-FLs. In previous reports, different kinds of multiple soliton operating states have been observed in the PML-FLs, including soliton bunches [36], bound state solitons [52], soliton collisions [53], vibration of soliton pairs [54], and restless solitons [55]. Based on the complex Ginzburg–Landau equation (CGLE), Malomed in 1991 [56] first pointed out that weakly stable two- and multi-bound states of solitons exist inside the laser cavity. Experimentally, the characteristics of bound state solitons in PML-FLs have been widely reported. For example, Zhao et al. [57] in 2004 reported bound states of twin-pulse solitons in PML-EDFL by the NPR because of the dispersive wave mediated long-range soliton interaction. By the proper adjusting of the pump power or the angle of PCs in the anomalous dispersion regime, more than one pulse appears in one cavity round trip due to quantization of the soliton energy [37, 58]. Lin et al. [59] in 2015 demonstrated that the multiple bound solitons and the bound states of the multiple dispersion-managed solitons occur in the net normal dispersion cavity of the PML Yb-doped fiber laser.

**Figure 6:**
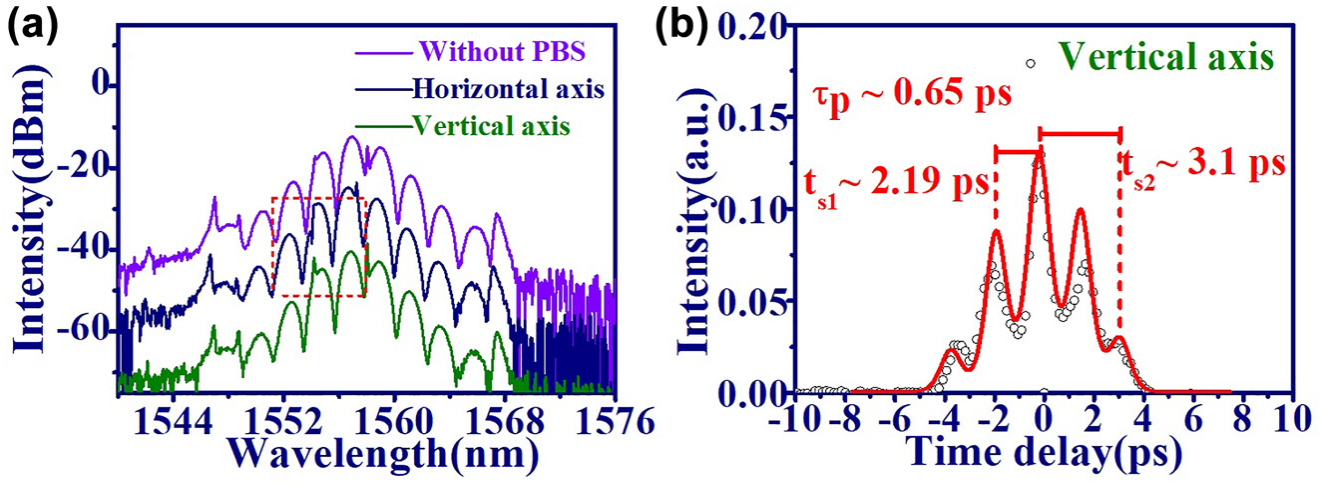
Bound GVLVSs from EDFL based on GO/PVA film. (a) Optical spectrum of (without PBS: purple line, horizontal axis: navy line and vertical axis: green line), (b) IAC trace on the vertical axis (open circle) and theoretical fitting curve (red solid line) by Eqs. (3) and (4).

In this work, bound state, bunch, and HML GVLVSs have also been observed in PML-EDFL. The optical spectrum of the bound GVLVSs is shown in Figure 6(a) (horizontal axis: navy, vertical axis: green, and without PBS: purple lines). The spectra reveal obvious amplitude modulation resulting from the interference of bound solitons. Besides, the peak wavelength from two orthogonal components (horizontal and vertical axes) of the GVLVSs shifts slightly and reveals peak-valley shapes inside the red dashed box. In Figure 6(b), the IAC trace (open circles) of bound state GVLVSs on vertical axis indicates that three solitons are bounded together. Theoretically, the measured IAC trace is ascribed by [59]

(3) $$I_{AC}\left(\tau \right)=\overset{\infty }{\underset{-\infty }{\int }}I\left(t\right)I\left(t-\tau \right)dt,$$

where *I*(*t*) = |*E*(*t*)|^2^ is the intensity, and *E*(*t*) is the electric field distribution. In assuming the hyperbolic secant function, the electric field of triple bound solitons is described by

(4) $$\begin{array}{ll} E\left(t\right) & =A_{1}sech\left[{\left(t/\left(\tau_{p}\times 1.762\right)\right)}^{2}\right]\\ & \;+A_{2}sech\left[{\left(\left(t-t_{s1}\right)/\left(\tau_{p}\times 1.762\right)\right)}^{2}\right]\\ & \;+A_{3}sech\left[{\left(\left(t+t_{s2}\right)/\left(\tau_{p}\times 1.762\right)\right)}^{2}\right], \end{array}$$

in which *A*_1_, *A*_2_, and *A*_3_ are scaling factors, *τ*_*p*_ is the pulsewidth of the mode-locked pulse, and *t*_*s*1_ and *t*_*s*2_ are the separation times of the triple bound solitons. By the theoretical fitting considering Eqs. (3) and (4), the well fitted intensity distribution of the IAC trace (red solid curve) for the triple bound solitons is shown in Figure 6(b). Here, the pulsewidth of the soliton is about 650 fs and the separation times *t*_*s*1_ and *t*_*s*2_ of the two bound solitons are about 2.19 and 3.10 ps, respectively.

**Figure 7:**
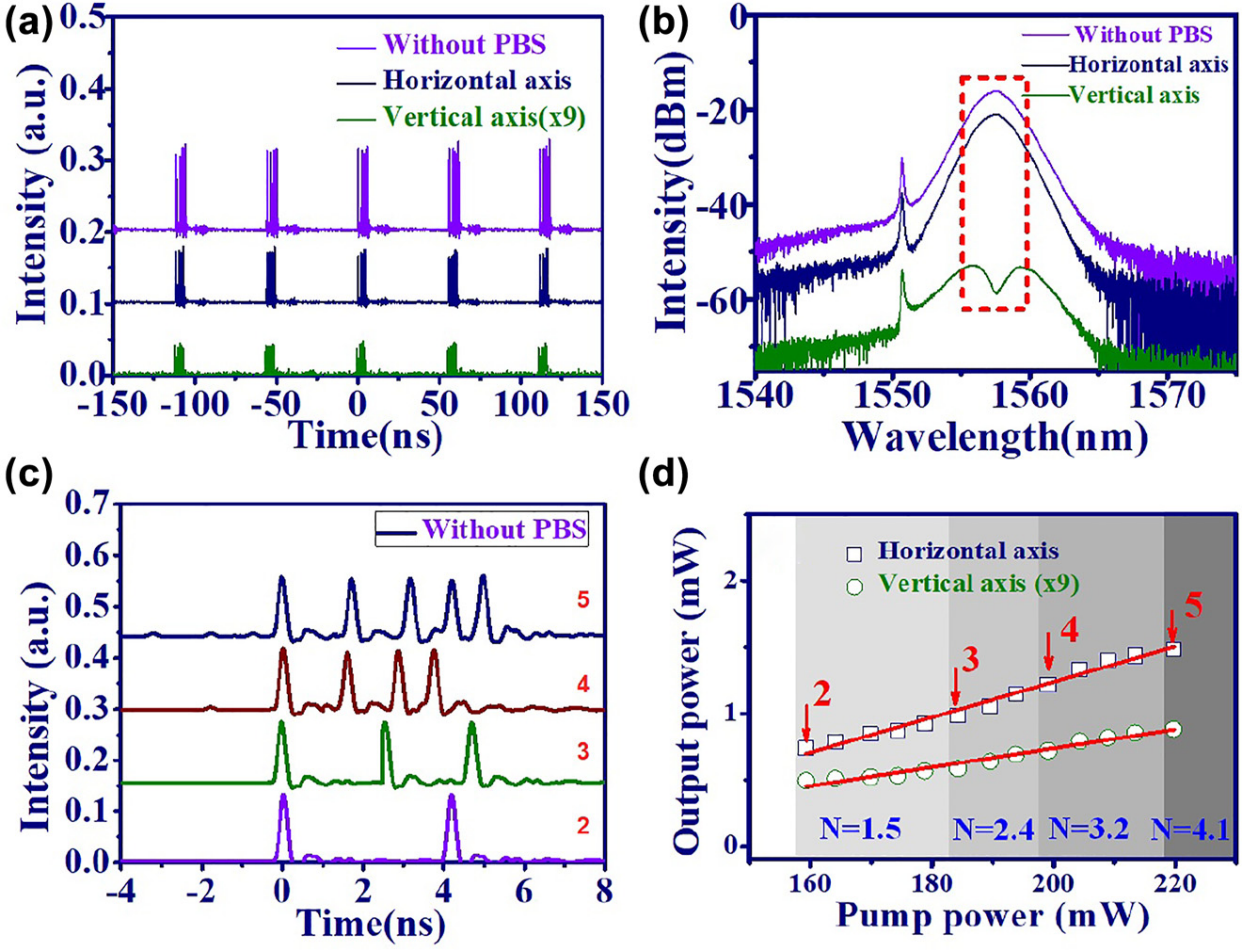
Bunch GVLVSs form PML-EDFL based on GO/PVA film. (a) Time trace and (b) optical spectrum of the quintuple GVLVSs (without PBS: purple line, horizontal axis: navy line and vertical axis: green line). (c) Time trace of different operation state bunch GVLVSs (purple line: the dual, green line: the triple, brown line: the quadruple and navy line: the quintuple vector solitons). (d) Output power of bunch GVLVSs as a function of pump power in operation in different states.

On the other hand, the time trace and optical spectrum of quintuple bunch GVLVSs (horizontal axis: navy, vertical axis: green, and without PBS: purple lines) are shown in Figure 7(a) and (b). The time trace of PML-EDFL indicates that five solitons coexist in each cavity round trip time. Unlike the typical emission spectrum in the horizontal axis (navy line) and without PBS (purple line), a double hump, i.e., central dip, emission spectrum is observed on the vertical axis (green line) for the corresponding spectrum in Figure 7(b). Furthermore, the dip on the vertical axis reveals a slight shift relative to the peak on the horizontal axis. Figure 7(c) shows the zoomed in time trace (without PBS) of the dual (purple line), triple (green line), quadruple (brown line), and quintuple (navy line) solitons. The output power of GVLVSs in operation in different states (horizontal axis: open square, vertical axis: open circle) as a function of pump power is shown in Figure 7(d). Theoretically [60], the quantization and number of solitons inside the cavity is determined by

(5) $$N=\sqrt{L_{D}/L_{NL}}=\tau_{p}\sqrt{\gamma P_{p}/|\beta_{2}|},$$

where *N* (nearest integer) is the number of solitons, $L_{D}=\frac{T_{0}^{2}}{\left|\beta_{2}\right|}$ is the dispersion length of the fiber, $L_{NL}=\frac{1}{\gamma P_{p}}$ is the nonlinear length, *τ*_*p*_ ≈ 0.79 ps is the pulsewidth of the mode-locked pulse, *P*_*p*_ is the peak power and *β*_2_ ≈ −0.144 ps^2^ is the group velocity dispersion. $\gamma =\frac{n_{2}\omega_{0}}{cA_{\text{eff}}}$ is the nonlinear-index coefficient, where *n*_2_ ≈ 3 × 10^−20^ m^2^/W is the nonlinear refractive index, *w*_0_ is the angular frequency, *c* is the speed of light and *A*_eff_ ≈ 1.96 × 10^−11^ m^2^ is the effective mode area. By Eq. (5), the solitons number *N* for dual, triple, and quadruple GVLVSs is estimated to be 1.5, 2.4, 3.2, and 4.1, which is consistent with the experimental result.

**Figure 8:**
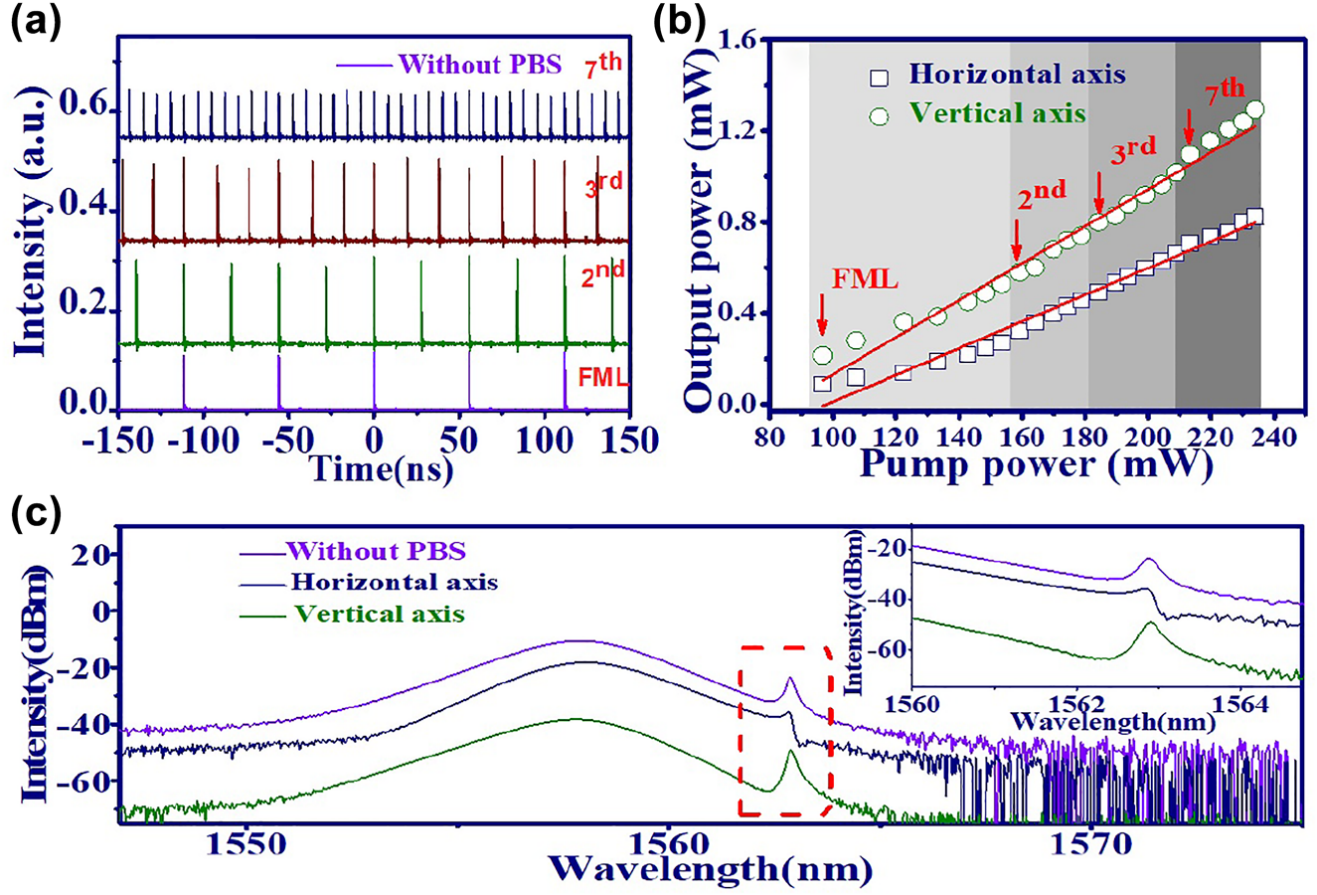
HML GVLVSs of EDFL based on GO/PVA film. (a) Time trace (FML: purple line, 2nd HML: green line, 3rd HML: brown line and 7th HML: navy line.), (b) output power of GVLVSs as a function of pump power in operation in different harmonic states, (c) optical spectrum of the 7th HML GVLVSs and zoomed in spectrum of the Kelly sidebands (inset figure).

For the laser in operation in the HML state [61–63], the split pulses in each cavity round trip time reveal an equal time interval between sequential pulses. Some mechanisms have been proposed to explain HML, such as soliton interaction by acoustic effects [25, 64] and the transient gain depletion and recovery dynamics in the gain medium [65, 66]. Experimentally, the HML from this PML-EDFL was achieved by adjusting PCs at certain pump power. Figure 8(a) shows the time trace of EDFL (without PBS) in operation at the FML (purple line), the 2nd (green line), the 3rd (brown line) and the 7th (navy line) HML state. For the *n*th high order HML state, the pulse repetition rate becomes *n* time of fundamental repetition rate. The output powers of HML GVLVSs (horizontal axis: open squares, vertical axis: open circles) as a function of pump power is shown in Figure 8(b). It is clear to see that the high order HML state will be generated at higher pump power [67, 68]. Figure 8(c) shows the optical spectrum (horizontal axis: navy line, vertical axis: green line, without PBS: purple line) of the 7th HML. The zoomed in spectrum inside the red dashed box indicates that the Kelly sidebands in the two orthogonal components reveal a peak-valley shape. In addition, the peak on the horizontal axis shows a slight blue shift around 0.1 nm relative to the peak on the vertical axis for the compensation of group velocity mismatch from the PMF. Table 1 lists the observed VSs dynamics by the GO/PVA film in this work for the comparison to other reports based on graphene.

**Table 1:** Vector solitons in Er-doped fiber laser based on 2D material SAs.

Materials	Modulation	Incorporation	Soliton type	Ref
	depth (%)	method		
Graphene	23	Fiber end facet	PLVSs, PRVSs, multiple PRVSs	[30]
Graphene	–	Fiber end facet	Bound VSs, bound state of bound VSs	[35]
Graphene	–	Fiber end facet	Bunch VSs, soliton rain PLVSs, soliton rain PRVSs	[36]
Graphene	–	Fiber end facet	PLVSs, PRVSs, bound state PLVSs, bound PRVSs, HML PLVSs	[37]
GO/PVA film	19.5	Sandwiched	Dual wavelength GVLVS, PL-GVLVS, PR- GVLVS bound GVLVSs, bunch GVLVSs, HML GVLVSs	Our work

## 4 Conclusions

In summary, we have investigated the versatile dynamic of group velocity locked vector solitons (GVLVSs) from passively mode-locked erbium-doped fiber laser (PML-EDFL) with insertion of a segment of polarization maintaining fiber (PMF) inside the cavity. Here, the GO/PVA film was used as saturable absorbers (SAs) for the mode-locked pulse generation, which reveals a 19.5% modulation depth and 1.21 MW/cm^2^ saturation intensity from nonlinear optical measurement. Generally, the Kelly sidebands of GVLVSs in the two orthogonal components reveal peak-valley or valley-peak alternation and slight shift of the emission peak for the compensation of group velocity mismatch. By means of the Lyot filter, we observed the dual wavelength GVLVSs in EDFL, in which the separation of the two emission peaks was determined by the insertion length of PMF. Unlike polarization locked GVLVSs showing almost fixed peak intensity, the polarization rotation GVLVSs reveal periodic intensity alternation between two orthogonal components. Furthermore, the period-doubling phenomenon has been revealed in their two orthogonal components but cannot be seen from the output of EDFL without passing through a polarization beam splitter. For the bound state GVLVSs, the spectrum reveals strong amplitude modulation from the interference of separated soliton molecules. For the bunch GVLVSs, a peculiar double hump spectrum was shown on the vertical axis. Consistence with the theoretical estimation, the number of solitons increases with the pump power. We also demonstrated the HML GVLVSs in this work, which showed the 7th harmonic mode locked state at highest pump power. All the observed results indicate that the EDFL based on GO/PVA film can be an excellent platform for studying peculiar phenomena of soliton dynamics.
